# A reliable multiplex genotyping assay for HCV using a suspension bead array

**DOI:** 10.1111/1751-7915.12140

**Published:** 2014-07-10

**Authors:** Yi-Chen Yang, Der-Yuan Wang, Hwei-Fang Cheng, Eric Y Chuang, Mong-Hsun Tsai

**Affiliations:** 1 Food and Drug Administration, Ministry of Health and WelfareTaipei, Taiwan; 2 Institute of Biotechnology, National Taiwan UniversityTaipei, Taiwan; 3 Graduate Institute of Biomedical Electronics and Bioinformatics, National Taiwan UniversityTaipei, Taiwan; 4 Department of Electrical Engineering, National Taiwan UniversityTaipei, Taiwan; 5 Center for Biotechnology, National Taiwan UniversityTaipei, Taiwan

## Abstract

The genotyping of the hepatitis C virus (HCV) plays an important role in the treatment of HCV because genotype determination has recently been incorporated into the treatment guidelines for HCV infections. Most current genotyping methods are unable to detect mixed genotypes from two or more HCV infections. We therefore developed a multiplex genotyping assay to determine HCV genotypes using a bead array. Synthetic plasmids, genotype panels and standards were used to verify the target-specific primer (TSP) design in the assay, and the results indicated that discrimination efforts using 10 TSPs in a single reaction were extremely successful. Thirty-five specimens were then tested to evaluate the assay performance, and the results were highly consistent with those of direct sequencing, supporting the reliability of the assay. Moreover, the results from samples with mixed HCV genotypes revealed that the method is capable of detecting two different genotypes within a sample. Furthermore, the specificity evaluation results suggested that the assay could correctly identify HCV in HCV/human immunodeficiency virus (HIV) co-infected patients. This genotyping platform enables the simultaneous detection and identification of more than one genotype in a same sample and is able to test 96 samples simultaneously. It could therefore provide a rapid, efficient and reliable method of determining HCV genotypes in the future.

## Introduction

Hepatitis C virus (HCV) is one of the leading causes of chronic hepatitis, liver cirrhosis and hepatocellular carcinoma. At least six major HCV genotypes have been identified worldwide, and the difference among the sequences of different genotypes is approximately 30% (Simmonds *et al*., 2005). HCV genotypes 1, 2 and 3 are globally distributed; genotype 4 is predominant in the Middle East, and genotypes 5 and 6 appear to be restricted to South Africa and Southeast Asia respectively (Zein, 2000). Several studies have found that HCV genotype 1b is associated with more severe liver disease and a more aggressive course than other HCV genotypes (Amoroso *et al*., 1998; Zein, 2000; Simmonds, 2004; Bostan and Mahmood, 2010). Furthermore, patients infected with different HCV genotypes might respond to interferon/ribavirin therapy differently; for example, genotypes 1 and 4 have exhibited more resistance than genotypes 2 and 3. This finding suggests that HCV genotype determination plays an important role in the clinical treatment strategy for HCV-infected patients (Simmonds, 2004; Simmonds *et al*., 2005; Chevaliez and Pawlotsky, 2009; Chevaliez, 2011). Recently, HCV genotype determination has been incorporated into the treatment guidelines for HCV infections, including the 2009 American Association for the Study of Liver Diseases guidelines and the 2011 European Association for the Study of the Liver clinical practice guidelines. According to these guidelines, it is very important to determine the HCV genotype or genotypes that are present prior to treatment to establish the duration of treatment, the ribavirin dose and the virological monitoring procedure (Ghany *et al*., 2009; EASL, 2011).

Several techniques have been developed for HCV genotyping, such as direct sequencing, DNA hybridization and multiplex real-time reverse transcription polymerase chain reaction (RT-PCR) (Weck, 2005; Al Olaby and Azzazy, 2011). Although direct sequencing is a gold standard for HCV genotyping, this approach is time consuming and not suitable for routine use in clinical laboratories. Furthermore, a significant limitation of direct sequencing for genotyping is its inability to determine two or more HCV genotypes within one sample (Zein, 2000; Bartholomeusz and Schaefer, 2004). The HCV Versant LiPA 2.0 assay, which is based on DNA hybridization, has been widely used in clinical laboratories. However, the assay failed to detect genotype 2 or misclassified four instances of genotype 1 as genotype 2 in 50 HCV-infected specimens (Scott and Gretch, 2007; Molenkamp *et al*., 2009). Another HCV genotyping assay based on real-time polymerase chain reaction (PCR) technology has recently been announced and commercialized (Abbott RealTime HCV Genotype II; Abbott Molecular, Abbott Park, IL, USA). This assay amplifies a portion of the HCV genome to determine the genotype in a single step by targeting the 5′ untranslated region (5′UTR) for genotypes 2a, 2b, 3, 4, 5 and 6 and the NS5B region for genotypes 1a and 1b. However, the assay has reportedly misclassified genotype 1 as genotype 6 on occasion. Furthermore, it is quite expensive and not suitable for routine use, which restricts its broad application (Martro *et al*., 2008). Thus, there is a need to develop a rapid, inexpensive and high-throughput genotyping assay.

Suspension bead arrays are three-dimensional arrays that employ beads as solid surface supports for various types of target-specific sequences. The targets are fluorescently labelled and hybridized to the target-specific sequences captured on the beads. Flow cytometry is then used for bead identification and target detection. The advantage of a suspension bead array is its ability to simultaneously detect and distinguish several targets in the same specimen with ease and accuracy. The convenience of universal bead sets and their flexibility makes the development of user-defined applications practical and comparatively inexpensive. The comparative ease, high multiplexing potential and affordability of suspension bead arrays make this platform the most attractive for high-throughput nucleic acid detection in clinical diagnostics (Miller and Tang, 2009; Deshpande and White, 2012).

The objectives of this study were to develop a reliable multiplex genotyping assay for HCV using a suspension bead array. In this study, the genotype-specific single nucleotide variants (GSNVs) of various HCV genotypes were identified, and target-specific primers (TSPs) were designed. This system can detect and identify more than one target in the same sample simultaneously, and it has the potential for employment in clinical microbiology laboratories in the future.

## Results

### Testing the genotype-specific TSPs designed for the HCV genotyping assay

**Figure 1:**
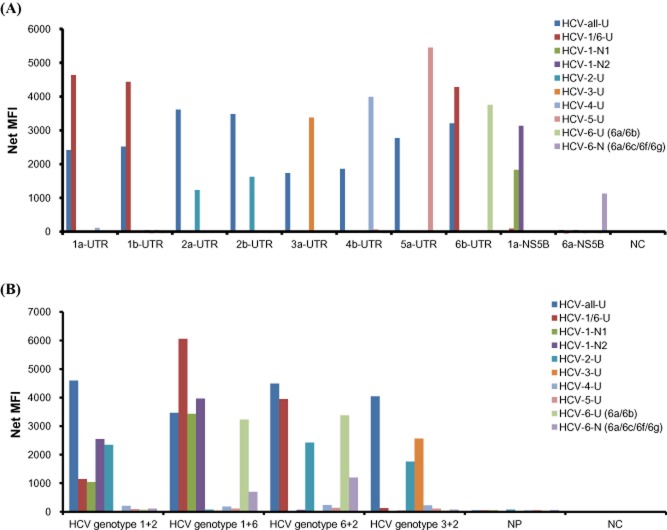
The genotype-specific TSPs could correctly detect and distinguish the genotypes present in both the specific-genotype plasmids and the mixed-genotype plasma samples.A. Synthetic plasmids of various HCV genotypes were used to test the specificity of each TSP designed for the assay.B. Artificially mixed-genotype plasma samples were used to evaluate the performance of the assay.Net MFI, net medium fluorescence intensity; NC, background control; NP, negative plasma control. The cut points of each genotype-specific bead: HCV-all-U (117.2); HCV-1/6-U (304.8); HCV-1-N1 (145.2); HCV-1-N2 (105.5); HCV-2-U (144.3); HCV-3-U (167.1); HCV-4-U (648.1); HCV-5-U (380); HCV-6-U (6a/6b) (130.5); and HCV-6-N (6a/6c/6f/6g) (123.4).

The TSPs were designed to detect either all genotypes or six different HCV genotypes by targeting the 5′UTR and NS5B regions. In these TSPs, HCV-all-U was designed to detect all genotypes, and HCV-1/6-U was designed to detect both genotypes 1 and 6 by targeting the 5′UTR region. In addition, HCV-1-N1 and HCV-1-N2 were designed to detect genotype 1 by targeting the NS5B region, and HCV-6-N was designed to detect genotype 6 by targeting the NS5B region. Plasmids carrying six different HCV genotypes were synthesized and used to test the suitability of these genotype-specific TSPs for the HCV genotyping assay. The results indicated that only HCV-all-U and HCV-1/6-U TSPs demonstrated significantly higher intensity for the 5′UTR plasmids of genotypes 1a and 1b. For the 5′UTR plasmid of genotype 6, only the HCV-all-U, HCV-1/6-U and HCV-6-U (6a/6b) TSPs exhibited significantly higher intensities. Because HCV-all-U was designed to detect all genotypes, these results confirmed the suitability of the genotype-specific TSPs. Similar results were also observed for the other genotype plasmids (Fig. 1A, Supporting Information Fig. S1A). In addition, various concentrations of genotype plasmids were used to evaluate the sensitivity of the assay, and the results indicated that the 5′UTR plasmid of genotype 6 could be detected in a dose-dependent manner at concentrations as low as 10^−6^ ng ml^–1^ (Supporting Information Fig. S1B). Similar results were also observed for the other genotype plasmids. Overall, the results indicated that the genotype-specific TSPs could correctly detect and distinguish their corresponding genotype plasmids.

### Sensitivity and specificity evaluations of the HCV genotyping assay

An HCV genotype 2 standard (TFDA code: 101-08) that was prepared from human plasma and assigned a value of 1.4 × 10^5^ IU ml^–1^ calibrated against the World Health Organization International Standard (NIBSC code: 06/102) as determined through a collaborative study was used to evaluate the sensitivity of this HCV genotyping array assay. As shown in Supporting Information Fig. S2A, only HCV-all-U and HCV-2-U exhibited significant and dose-dependent signals. The results revealed that the assay could correctly determine the HCV genotype with a detection limit of approximately 10^2^–10^3^ IU ml^–1^.

Several blood-borne virus standards, including the hepatitis A virus (HAV), hepatitis B virus (HBV), human immunodeficiency virus (HIV)-1 and parvovirus B19 (B19V) standards, were used to evaluate the analytic specificity of the HCV genotyping assay. As shown in Supporting Information Fig. S2B, no significant high-intensity signals were observed in the samples without the HCV standard. Only the samples spiked with 10^4^ IU ml^–1^ HCV standard (TFDA code: 93-09) exhibited significant signals of the correct type. The detection and discrimination capabilities of the assay were not affected by the presence of other blood-borne pathogens, such as HAV, HBV, HIV-1 or B19V, indicating that the assay is highly specific and robust.

The HCV RNA genotype performance panel was used to evaluate the performance of the HCV genotyping assay. The panel set was prepared from various genotypes collected from plasma samples from HCV-infected patients. As shown in Table 1, the results demonstrated that the genotype-specific TSPs could correctly detect and distinguish the different genotypes of the panel samples, suggesting that the assay could correctly identify genotypes in HCV-infected patients.

**Table 1:** A BBI genotype panel was used to evaluate the reliability of the high-throughput HCV genotyping system based on a suspension bead array

			Results for each genotype-specific TSP									
Panel member	Genotype	Interpretation	HCV-all-U	HCV-1/6-U	HCV-1-N1	HCV-1-N2	HCV-2-U	HCV-3-U	HCV-4-U	HCV-5-U	HCV-6-U (6a/6b)	HCV-6-N (6a/6c/6f/6g)
PHW203-01	Genotype 1	Genotype 1	+	+	+	+	−	−	−	−	−	−
PHW203-02	Genotype 1	Genotype 1	+	+	+	+	−	−	−	−	−	−
PHW203-03	Negative plasma	−	−	−	−	−	−	−	−	−	−	−
PHW203-04	Genotype 2	Genotype 2	+	−	−	−	+	−	−	−	−	−
PHW203-05	Genotype 3	Genotype 3	+	−	−	−	−	+	−	−	−	−
PHW203-06	Genotype 4	Genotype 4	+	−	−	−	−	−	+	−	−	−
PHW203-07	Genotype 4	Genotype 4	+	−	−	−	−	−	+	−	−	−
PHW203-08	Genotype 5	Genotype 5	+	−	−	−	−	−	−	+	−	−
PHW203-09	Genotype 6a/6b	Genotype 6a/6b	+	+	−	−	−	−	−	−	+	+

### Clinical evaluation of the HCV genotyping assay

Thirty-five clinical samples (anti-HCV-positive plasma) without genotype information were used to evaluate the performance of the HCV genotyping assay. Nine samples were reported as ‘genotype 1’, sixteen samples were reported as ‘genotypes 2’, three samples were reported as ‘genotype 6’, four samples were reported as ‘genotype 1 or 6’ and three samples were reported as ‘no HCV’ according to the HCV genotyping assay. These results were compared with those of current genotyping methods, including direct sequencing and the Abbott RealTime HCV Genotype II assay (GT II kit; Abbott Molecular). According to the direct-sequencing results, 32 of the 35 samples were accurately genotyped by the array method (32/35, 91%), which was more accurate than the GT II kit (29/35, 83%). In the case of the array assay, the remaining three samples (one ‘genotype 1’ sample and two ‘genotype 6’ samples) were reported as ‘HCV genotype 1 or 6’, and the direct-sequencing data indicated that the two genotype 6 samples belonged to genotype 6n. By contrast, there were five samples (corresponding to genotypes 2 and 6) reported as ‘HCV indeterminate’ by the GT II kit. For the ‘genotype 6’ sample determination, there was one sample reported as ‘genotype 6 (reactivity with genotype 1)’ by the GT II kit (Table 2A). Overall, the results demonstrated that all samples could be successfully determined by the array assay, and its results approximately identical to those determined via direct sequencing. This finding strongly supported the reliability of this high-throughput genotyping system for HCV assays using a liquid microarray.

Because it is difficult to collect plasma samples from patients infected with different genotypes, several mixed-genotype plasma samples were prepared to evaluate the robustness of the HCV genotyping array assay. The results indicated that all mixed-genotype plasma samples were detected and the genotypes were correctly distinguished by the HCV genotyping assay for mixing ratios of 1:1 (Fig. 1B), 1:10 and 1:50. By contrast, only one of the four mixed-genotype plasma samples (genotypes 1 + 2) was detected with the correct genotype identification by the GT II kit for mixing ratios of 1:1, 1:10 and 1:50. The GT II kit failed to identify genotype 6 in a 1:1 mixed plasma of genotypes 1 + 6 at 10^4^ IU ml^–1^, and it failed to identify genotype 6 in 1:10 and 1:50 mixed plasma of genotypes 6 + 2 at 10^4^ IU ml^–1^. It also failed to identify either genotype 2 or 3 in 1:1, 1:10 and 1:50 mixed plasma of genotypes 3 + 2 at 10^4^ IU ml^–1^ (Table 2B). These results suggested that the assay we developed could correctly detect and distinguish between two different genotypes in mixed-genotype HCV plasma. Thus, this assay could be used to correctly identify different HCV genotypes in patients with multiple infections, e.g. hemophiliac patients and injection drug users (IDUs).

**Table 2:** A comparison of the HCV genotyping results obtained using the developed HCV genotyping assay and other methods for (A) clinical samples and (B) artificially mixed-genotype samples

(A) Clinical samples			
Anti-HCV plasma	Direct sequencing	HCV genotyping bead array assay	GT II kit
HCV genotype 1	10	9	11
HCV genotype 2	16	16	13
HCV genotype 6	5	3	2
HCV unclassified genotype	1^a^	4^b^	6^c^
ND	3	3	3
Total	35	35	35

a The genotype of one sample could not be confirmed via sequence analysis of the core and NS5B regions because the core and NS5B regions of the sample could not be amplified and sequenced.

b Four samples were reported as ‘genotype 1 or 6 (non 6a/6b)’.

c Five samples were reported as ‘HCV indeterminate’, and one sample was reported as ‘genotype 6 (reactivity with genotype 1)’.

**Table:** 

**(B) Artificially mixed-genotype samples**			
**Mixed-genotype samples**		**HCV genotyping bead array assay**	**GT II kit**
Genotypes 1 + 2	1:1 (1 × 10^4^ + 1 × 10^4^ IU ml^–1^)	Genotypes 1 + 2	Genotypes 1 + 2
	1:10 (2 × 10^4^ + 2 × 10^5^ IU ml^–1^)	Genotypes 1 + 2	Genotypes 1 + 2
	1:50 (2 × 10^4^ + 1 × 10^6^ IU ml^–1^)	Genotypes 1 + 2	Genotypes 1 + 2
Genotypes 1 + 6	1:1 (2 × 10^4^ + 2 × 10^4^ IU ml^–1^)	Genotypes 1 + 6	Genotype 1
	1:10 (2 × 10^4^ + 2 × 10^5^ IU ml^–1^)	Genotypes 1 + 6	Genotypes 1 + 6
	1:50 (2 × 10^4^ + 1 × 10^6^ IU ml^–1^)	Genotypes 1 + 6	Genotypes 1 + 6
Genotypes 6 + 2	1:1 (2 × 10^4^ + 2 × 10^4^ IU ml^–1^)	Genotypes 2 + 6	Genotypes 2 + 6
	1:10 (2 × 10^4^ + 2 × 10^5^ IU ml^–1^)	Genotypes 2 + 6	Genotype 2
	1:50 (2 × 10^4^ + 1 × 10^6^ IU ml^–1^)	Genotypes 2 + 6	Genotype 2
Genotypes 3 + 2	1:1 (1 × 10^4^ + 1 × 10^4^ IU ml^–1^)	Genotypes 2 + 3	Genotype 3 (reactivity with 2^a^)
	1:10 (2 × 10^4^ + 2 × 10^5^ IU ml^–1^)	Genotypes 2 + 3	Genotype 3 (reactivity with 2^a^)
	1:50 (2 × 10^4^ + 1 × 10^6^ IU ml^–1^)	Genotypes 2 + 3	Genotype 2

a According to the package insert of the GT II kit (pages 7–9 of the package insert, 51-602215/R4, 2011), the assay detected HCV and produced a genotype result (i.e. genotype 3) with an interpretation of reactivity with another genotype (i.e. genotype 2). This means that an additional genotype signal was observed (i.e. genotype 2). Based on known cross-reactive patterns and the knowledge that mixed infections are rarely observed, the additional genotype was interpreted to be reactive but was not included in the results column.

## Discussion

In this study, the assay system included 10 specific TSPs for six different HCV genotypes and two sets of primers targeting two of the most commonly used regions for HCV detection and genotyping. The primary advantage of this method is that the required sample volume is reduced to 0.2 ml as a result of the reliable classification offered by the assay for six HCV genotypes in parallel. For the same reason, the time and cost of analysis are also reduced.

The evaluation of genotype-specific plasmids indicated that the discrimination of the six genotypes by the TSPs designed for the HCV genotyping assay is extremely powerful. The sensitivity of the results demonstrated that this assay could detect and determine HCV genotype plasmids at 10^−6^ ng. Moreover, a similar result using an HCV standard indicated a limit of detection for HCV of approximately 10^2^–10^3^ IU ml^–1^. For a 0.2 ml sample volume, our assay exhibited similar sensitivity to the GT II kit, which claims limits of detection of 500 IU ml^–1^ for a 0.5 ml sample volume (used in a comparative study) and 1250 IU ml^–1^ for a 0.2 ml sample volume (Supporting Information Fig. S2A). This limit of detection may explain why some samples were reported with an ambiguous result of ‘HCV genotype 1 or 6’ (Table 2A) by our assay because of the low viral load. One such sample was quantified using the Cobas Ampliprep/Cobas TaqMan HCV test (CAP/CTM, Roche Diagnostic, Mannheim, Germany), and the concentration was as low as 100 IU ml^–1^, below the detectable limit. To precisely test plasma samples with viral loads lower than 500 IU ml^–1^, we could improve the sensitivity of the assay by using nested PCR instead of single PCR to increase the amplified region for bead detection.

More than 30 clinical specimens were included in this study to perform a comparative study with an Abbott GT II kit. Compared with direct sequencing, the results indicated that 91% of the samples were correctly determined by the HCV genotyping assay, and only 83% of the samples were correctly determined by the GT II kit. Six samples were not correctly classified by the GT II kit, and five of these six samples were reported as ‘HCV indeterminate’. Among these indeterminate samples, three samples were sequenced as genotype 2 and were correctly determined by our HCV genotyping assay. One potential reason for the indeterminate results may be that the sequences of these specimens included variations in the target region for the genotype 2 probe of the GT II kit (Hong *et al*., 2012). A similar situation has been described in the ‘Summary of Safety and Effectiveness Data (SSED) of the Abbott RealTime HCV Genotype II’ from the US Food and Drug Administration (FDA), in which four of 116 HCV genotype 2 samples were identified as ‘HCV detected, no genotype result’ and one sample was identified as ‘HCV not detected’.

HCV genotype 6 is predominantly located in Southeast Asia, and previous studies have indicated that genotype 6 is the most common genotype in Vietnam and Myanmar, with a prevalence rate of approximately 50%. In Taiwan, HCV genotype 6 is less common compared with genotypes 1 and 2, and it has replaced genotype 2 as the second most common genotype among IDUs (Lee *et al*., 2007; Chao *et al*., 2011; Fu *et al*., 2011). However, it is very difficult to correctly identify genotype 6 using most commercially available genotyping assays, primarily because the 5′UTR sequences of genotype 6 are almost identical to those of genotype 1 (Simmonds *et al*., 2005; Ross *et al*., 2007; Yu and Chuang, 2009). Consequently, older tests that target only the 5′UTR region for genotype determination may misclassify HCV genotype 6 as genotype 1 (Lee *et al*., 2007). The GT II kit, which was designed to determine the HCV genotype by targeting the 5′UTR region and to distinguish genotype 1a from 1b by targeting the NS5B region, could potentially misclassify genotype 1 isolates as genotype 6 (Weck, 2005; Martro *et al*., 2008). In this study, the results demonstrated that the GT II kit was unable to correctly classify three out of five samples of genotype 6. Two of the three samples were reported as ‘HCV indeterminate’, and one was reported as ‘genotype 6 (reactivity with genotype 1)’. These results were consistent with the evaluation results described in the package insert of the GT II kit (page 8 of the package insert, 51-602215/R4, 2011), which indicated that the 5′UTR genotype 1 probe of the kit cross-reacts with genotype 6b samples and other non-6a/6b samples of genotype 6. The data presented in the package insert of the GT II kit also indicated that the assay was able to correctly identify only 8 of 12 genotype 6 specimens tested (page 43 of the package insert, 51-602215/R1, 2008) or 10 of 12 genotype 6 specimens tested (page 8 of the package insert, 51-602215/R4, 2011). The Abbott GT II kit was recently approved by the US FDA for identifying patient HCV genotypes and aiding health-care professionals in determining appropriate treatment approach. However, we note that in the application for the in vitro diagnostics license in the United States, it was claimed only that the kit was able to differentiate genotypes 1, 1a, 1b, 2, 3, 4 and 5; genotype 6 was not included. Although the NS5B region is employed for subtype determination in the GT II kit, it can be used only to distinguish between genotypes 1a and 1b. By contrast, our assay identifies specific variants located in both the 5′UTR and NS5B regions for discriminating both genotypes 1 and 6. This study revealed that as a result, our assay distinguishes genotype 6 from genotype 1 more accurately and reliably than the GT II kit.

In this study, one sample was detected by the probe (genotype 1) targeting the 5′UTR region but not by the probes (genotypes 1a and 1b) targeting the NS5B region in the GT II kit; therefore, it was reported as ‘HCV genotype 1’ by the kit. The sample was similarly detected by the probes (HCV-all-U, HCV-1/6-U) targeting the 5′UTR region but not by the probes targeting the NS5B region in our HCV genotyping assay; therefore, it was reported as ‘HCV genotype 1 or 6’ by our assay. The sequencing data indicated that this sample might contain a new HCV variant because there was sequence information available only for the 5′UTR region, and the NS5B region of the sample could not be amplified for further sequencing. An additional study is ongoing to confirm the genotype of this new HCV variant. Because previous studies have indicated rapid sequence drift of HCV (Simmonds, 2004), we designed one TSP (HCV-all-U) targeting for all HCV genotypes in this array assay to overcome the problem of insufficient detection caused by the high mutation rate of HCV. The results of this study demonstrated that although this sample does not belong to a classical genotype 1 or 6, even this new HCV variant could still be identified as HCV by our assay.

Multiple HCV infections have been reported in some risk groups with repeated HCV exposures, e.g. hemophiliac patients and IDUs. Prevalence studies have suggested that the percentage of subjects with mixed HCV infections is high in these risk groups for repeated HCV exposures, but the reported prevalence varies from 0% to 25%. This wide range of infection percentages can be attributed to the different genotyping methods used in the different study groups (Kao *et al*., 1994; Preston *et al*., 1995; Pham *et al*., 2010) or to low sensitivities for the detection of mixed genotyping (Bowden *et al*., 2005). Currently available genotyping assays, especially direct sequencing, are designed to amplify and determine the predominant genotype in a sample. Consequently, genotypes at lower concentrations in a mixed sample could fail to be identified or could be misidentified (Buckton *et al*., 2006). HCV treatment requires knowledge of all HCV genotypes present, including the minor genotype in a mixed-genotype sample, to prevent unexpected impacts of treatment. Cloning and sequencing the HCV cDNA is an accurate approach for the detection of mixed-genotype infections. However, this method requires considerable effort and is not appropriate for routine use (Hu *et al*., 2000). For this reason, new assays that enable the determination of mixed genotypes at a high-throughput sample scale have become increasingly desirable. In this study, the results revealed that our assay is capable of simultaneously detecting two different genotypes within a sample. By contrast, the GT II kit was unable to distinguish between genotypes in several mixed-genotype plasma samples. A previous study has also demonstrated that the GT II kit was unable to detect some genotypes in a mixed infection in which the ratio of the viral load of the minority genotype to that of the dominant genotype was less than 1:100 (Martro *et al*., 2008).

Furthermore, because of the similar routes of viral transmission, HCV co-infection with HBV and/or HIV is commonly observed, especially in hemophiliac patients and IDUs (Soriano *et al*., 2006; Koziel and Peters, 2007). Approximately 25% of HIV-infected individuals in the Western world reportedly have a chronic HCV infection, and approximately 6% of HIV-positive individuals fail to develop HCV antibodies. Therefore, HCV RNA should be evaluated in HIV-positive individuals with unexplained liver disease and anti-HCV-negative results (Ghany *et al*., 2009). The results presented in Supporting Information Fig. S2 indicate that no signals were observed in the blood-borne virus samples without HCV. Only the samples spiked with a 10^4^ IU ml^–1^ HCV standard exhibited significant signals of the correct type. In addition, the ability of our assay to detect and discriminate HCV was not affected by the presence of other blood-borne viruses. These results suggest that the assay could correctly identify HCV in HCV/HIV or HCV/HBV co-infected patients. Furthermore, because the bead array system offers powerful discrimination among similar sequences in HCV genotypes in combination with flexibility and the possibility of expansion, it would be very easy to design specific TSPs for HBV and HIV detection and to incorporate them into our HCV genotyping assay, and doing so may be advantageous because of the high prevalence of HCV/HIV and HCV/HBV co-infection. The sample size of this platform is also flexible, thus enabling us to detect and identify the genotypes of up to 96 samples simultaneously. Automated nucleic acid extraction systems have typically been applied to increase the sample size for high-throughput applications. We found that the data from an automatic nucleic acid extraction system were similar to the data from the high pure viral nucleic acid kit (data not shown). This finding suggested that our assay could be easily adapted to an automated nucleic acid extraction system, not only for high-throughput applications but also to reduce the risk of amplicon contamination.

Some low-density microarray systems have been developed to identify different HCV genotypes, but samples with mixed infections were not considered during the clinical evaluations of these systems. Moreover, the low sample throughput and cost of these assays limit their breadth of application, especially in clinical use (Gryadunov *et al*., 2010). Suspension bead arrays can overcome these limitations (Miller and Tang, 2009), thereby reducing labor requirements and sample processing. In addition, the estimated cost per sample of our assay is approximately $45, which is substantially cheaper than the $114 cost per sample associated with the GT II kit. The low cost of our assay will facilitate its widespread use, especially in developing countries. An HCV genotyping method based on a suspension bead array has been previously reported (Duarte *et al*., 2010); however, this method has been demonstrated to identify only HCV genotypes 1, 2 and 3, and it does not satisfy global requirements, especially in the Middle East, South Africa and Southeast Asia. Moreover, our assay, which we developed on the Luminex platform, can be easily ported to other platforms, such as the VeraCode platform (Illumina, USA) (data not shown) or other commercially available platforms.

In conclusion, the HCV genotyping assay we developed is a powerful method for rapid, high-throughput and accurate identification of different HCV genotypes. This platform enables the simultaneous detection and identification of genotypes in up to 96 samples, and it could represent a rapid, efficient and reliable method of determining HCV genotypes in the future.

## Experimental procedures

### Primer and TSP designs

The conserved regions of HCV, 5′UTR, were selected for the primer and TSP designs. After 794 HCV sequences were collected from NCBI, a multiple-sequence alignment of the 5′UTR regions was performed using the CLC Main Workbench software (CLC Bio, Denmark). The GSNVs located in the conserved region were first identified, and the TSPs were then designed on the basis of these GSNVs for the various HCV genotypes. The NS5B region of HCV was selected to distinguish genotype 1 from genotype 6. Moreover, two common primer sets (Supporting Information Table S1) covering all genotype-specific TSPs located in the 5′UTR and NS5B regions (Supporting Information Table S1) were designed using the VeraCode Assay Designer software (Illumina).

### HCV genotyping array assay protocol

#### RNA extraction and multiplex RT-PCR

HCV RNA was isolated using a high pure viral nucleic acid kit or a MagNA pure compact nucleic acid isolation kit I (Roche, Mannheim, Germany) in accordance with the manufacturer's instructions. A background control (NC) and a negative plasma control (NP) containing RNase-free water and HCV-negative plasma, respectively, instead of HCV-infected plasma, were included in each assay run. Reverse RNA transcription was performed using a SuperScript III First-Strand Synthesis System for RT-PCR (Invitrogen, Carlsbad, USA) in accordance with the manufacturer's instructions. Multiplex PCR was performed with 10 μl of cDNA using a Qiagen Multiplex PCR kit (Qiagen, Hilden, Germany). The reaction consisted of 25 μl of multiplex PCR master mix, 5 μl of Q solution and 0.2 μM of each primer. The PCR conditions were as follows: 95°C for 15 min and 40 cycles at 94°C for 30 s, 48.5°C for 90 s and 72°C for 90 s.

#### Target-specific primer extension (TSPE)

The multiplex PCR products were then treated with ExoSAP-IT reagent (USB, Ohio, USA) as follows: a mixture of 7.5 μl of PCR product and 3 μl of ExoSAP-IT reagent was incubated at 37°C for 30 min and at 80°C for 15 min. Five microlitres of each ExoSAP-IT-treated PCR product was then added to a 20 μl reaction solution containing 15–50 nM of each sequence-tagged TSP (Supporting Information Table S1) with 5 μM of each dNTP except dCTP, 5 μM of biotin-dCTP (Invitrogen Life Technologies, Carlsbad, CA, USA), 1.25 mM MgCl_2_ and 0.75 U of platinum Tsp Taq DNA polymerase (Invitrogen Life Technologies). The samples were cycled under the following conditions: 96°C for 2 min and 40 cycles at 94°C for 30 s, 50°C for 30 s and 72°C for 90 s.

#### Bead array hybridization and analysis

Ten different bead sets with specific sequences were combined in a bead mixture containing 2500 beads from each set. After centrifugation for 5 min, the beads were re-suspended in Tm hybridization buffer, and 25 μl of the suspension was dispensed into each well of a 96-well plate. Then, 5 μl of capture-sequence-tagged TSPE product was added to the bead mixture in each well. The 96-well plate was incubated at 95°C for 5 min for denaturation and was then hybridized at 37°C for 30 min. The coupled microspheres were then collected and re-suspended in 75 μl of reporter solution (10 μg ml^–1^ streptavidin-conjugated phycoerythrin in hybridization buffer) to incubate at 37°C at 250 r.p.m. for 15 min. The 96-well plates were then analysed by Luminex 200 system (Abbott). A minimum of 100 counts was recorded for each bead set. The medium fluorescence intensity (MFI) was obtained for each sample. The net MFI represents the MFI minus the NC. A positive result was defined as a net MFI greater than the cut-point value for each genotype-specific bead. The cut point for each genotype-specific bead was generated from the ROC curve analysis (Swets, 1988; Hajian-Tilaki, 2013), which was based on an analysis of 380 data points for each bead type.

### Testing the genotype-specific TSPs using synthesized HCV genotype plasmids

To test the specificity of the genotype-specific TSPs, the 5′UTR and NS5B sequences from various HCV genotypes were collected from NCBI (1a: EU255953; 1b: AB442222; 2a: NC009823; 2b: AY232738; 3: AF046866; 4; FJ462435; 5: NC009826 and 6: NC_009827). The sequences were synthesized and cloned into plasmids (TOP pUC57), which were then transformed into the *Escherichia coli* strain TOP10 (GenScript, USA). Plasmid DNA was extracted using a QIAprep Spin Miniprep Kit (QIAGEN, Hilden, Germany) for further HCV genotyping.

### Sensitivity and specificity evaluation

To evaluate the analytic specificity and sensitivity of our HCV genotyping assay, several blood-borne virus standards and an HCV genotype panel were used, including the HAV International standard (NIBSC code: 00/560), the HBV national standard (TFDA code: 92-08), HCV national standards for genotype 1 (TFDA code: 93-09) and genotype 2 (TFDA code: 101-08), the HIV-1 national standard (TFDA code: 98-11), the B19V national standard (TFDA code: 94-08) and the HCV RNA Genotype Performance Panel (BBI, PHW203). Nucleic acid extraction, multiplex RT-PCR, TSPE, bead array hybridization and analysis were performed following the procedures described previously.

### Clinical evaluation

To validate the performance of the HCV genotyping assay, 35 anti-HCV-positive plasma samples were analysed. In addition, 12 samples with two different artificially mixed genotypes were used to evaluate the robustness of the assay. These mixed-genotype plasma samples were prepared from randomly selected concentrations of two types of HCV, including genotypes 1, 2, 3 and 6. Nucleic acid extraction, multiplex RT-PCR, TSPE, bead array hybridization and analysis were performed following the procedures described previously.

The results were compared with those of current genotyping methods, including direct sequencing and the GT II kit. The target regions for direct sequencing were 5′UTR and NS5B. The GT II kit was applied in accordance with the manufacturer's instructions in conjunction with Abbott's m2000 automated real-time PCR platform (m2000sp and m2000rt).

## Supporting Information

**Fig. S1.** Evaluate the specificity and sensitivity using synthetic plasmids. A. Synthetic plasmids of different HCV genotypes were used to test the specificity of each TSP designed in the assay. B. Serial dilutions corresponding to 10^-3^, 10^-4^, 10^-5^, 10^-6^, 10^-7^ and 10^-8^ ng of the 5'UTR plasmid for HCV genotype 6 were used to perform the sensitivity evaluation of the HCV genotyping array assay. Net MFI: net medium fluorescence intensity; NC: background control. The cut point of each genotype-specific bead: HCV-all-U [117.2]; HCV-1/6-U [304.8]; HCV-1-N1 [145.2]; HCV-1-N2 [105.5]; HCV-2-U [144.3]; HCV-3-U [167.1]; HCV-4-U [648.1]; HCV-5-U [380]; HCV-6-U (6a/6b) [130.5]; HCV-6-N (6a/6c/6f/6g) [123.4]. **Fig. S2.** Evaluate the specificity and sensitivity using the blood-borne virus standards. A. Serial dilutions corresponding to 10^5^, 10^4^, 10^3^, 10^2^ and 10^1^ IU/mL of HCV genotype 2 standard (TFDA code: 101-08) were used to evaluate the analytical sensitivity of this HCV genotyping array assay. B. Several blood-borne virus standards, including HAV international standard (NIBSC code: 00/560), HBV national standard (TFDA code: 92-08), HCV genotype 1 standard (TFDA code: 93-09), HIV-1 national standard (TFDA code: 98-11) and B19V national standard (TFDA code: 94-08), were diluted to 10^4^ IU/mL and used to evaluate the analytical specificity of this HCV genotyping array assay. Net MFI: net medium fluorescence intensity; NP: negative plasma control; NC: background control. The cut point of each genotype-specific bead: HCV-all-U [117.2]; HCV-1/6-U [304.8]; HCV-1-N1 [145.2]; HCV-1-N2 [105.5]; HCV-2-U [144.3]; HCV-3-U [167.1]; HCV-4-U [648.1]; HCV-5-U [380]; HCV-6-U (6a/6b) [130.5]; HCV-6-N (6a/6c/6f/6g) [123.4].

**Table S1.** The primers and target-specific primers used for the HCV genotyping assay.
