# Supplementary material for: A reliable multiplex genotyping assay for HCV using a suspension bead array
Source: Microb Biotechnol. 2014 Jul 10;8(1):93–102. doi: 10.1111/1751-7915.12140 (PMC4321376; doi:10.1111/1751-7915.12140)
Supplement: Supplementary file 1 [file mbt20008-0093-sd1.pdf]

Figure legends

Figure S1. Evaluate the specificity and sensitivity using synthetic plasmids. (A) Synthetic plasmids of different HCV genotypes were used to test the specificity of each TSP designed in the assay. **(B)** Serial dilutions corresponding to 10^{-3}, 10^{-4}, 10^{-5}, 10^{-6}, 10^{-7}, and 10^{-8} ng of the 5'UTR plasmid for HCV genotype 6 were used to perform the sensitivity evaluation of the HCV genotyping array assay. Net MFI: Net Medium fluorescence intensity; NC: background control. The cut point of each genotype-specific bead: HCV-all-U [117.2]; HCV-1/6-U [304.8]; HCV-1-N1 [145.2]; HCV-1-N2 [105.5]; HCV-2-U [144.3]; HCV-3-U [167.1]; HCV-4-U [648.1]; HCV-5-U [380]; HCV-6-U (6a/6b) [130.5]; HCV-6-N (6a/6c/6f/6g) [123.4].

Figure S2. Evaluate the specificity and sensitivity using the blood-borne virus standards. (A) Serial dilutions corresponding to 10^5, 10^4, 10^3, 10^2, and 10^1 IU/mL of HCV genotype 2 standard (TFDA code: 101-08) were used to evaluate the analytical sensitivity of this HCV genotyping array assay. **(B)** Several blood-borne virus standards, including HAV International standard (NIBSC code: 00/560), HBV national standard (TFDA code: 92-08), HCV genotype 1 standard, (TFDA code: 93-09), HIV-1 national standard (TFDA code: 98-11), and B19V national standard (TFDA code: 94-08) were diluted to 10^4 IU/mL and used to evaluate the analytical specificity of this HCV genotyping array assay. Net MFI: Net Medium fluorescence intensity; NP: negative plasma control; NC: background control. The cut point of each genotype-specific bead: HCV-all-U [117.2]; HCV-1/6-U [304.8]; HCV-1-N1 [145.2]; HCV-1-N2 [105.5]; HCV-2-U [144.3]; HCV-3-U [167.1]; HCV-4-U [648.1]; HCV-5-U [380]; HCV-6-U (6a/6b) [130.5]; HCV-6-N (6a/6c/6f/6g) [123.4].

Figure S1.

(A)

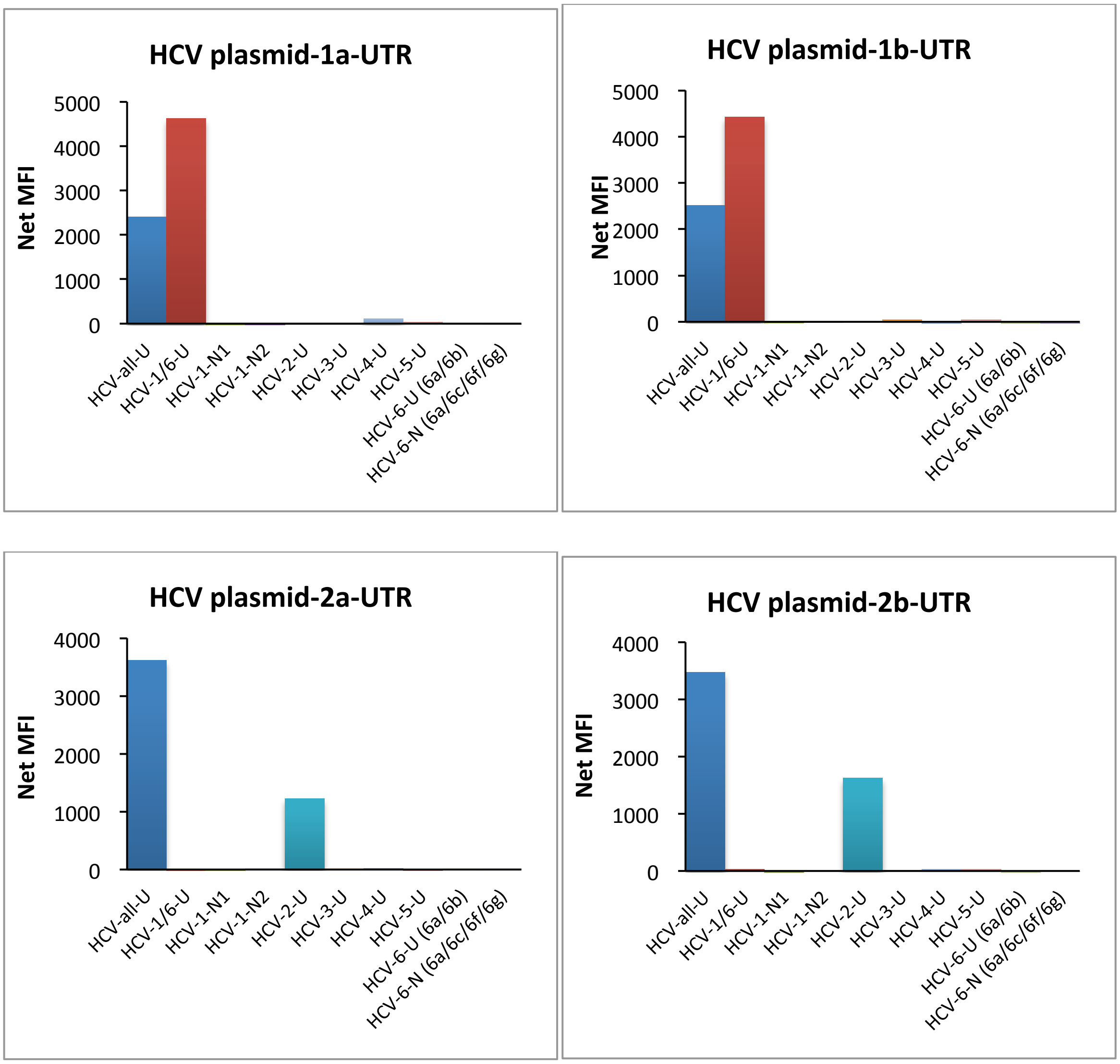
HCV plasmid-1a-UTR
HCV plasmid-1b-UTR
HCV plasmid-2a-UTR
HCV plasmid-2b-UTR
Net MFI
0
1000
2000
3000
4000
5000
HCV-all-U
HCV-1/6-U
HCV-1-N1
HCV-1-N2
HCV-2-U
HCV-3-U
HCV-4-U
HCV-5-U
HCV-6-U (6a/6b)
HCV-6-N (6a/6c/6f/6g)

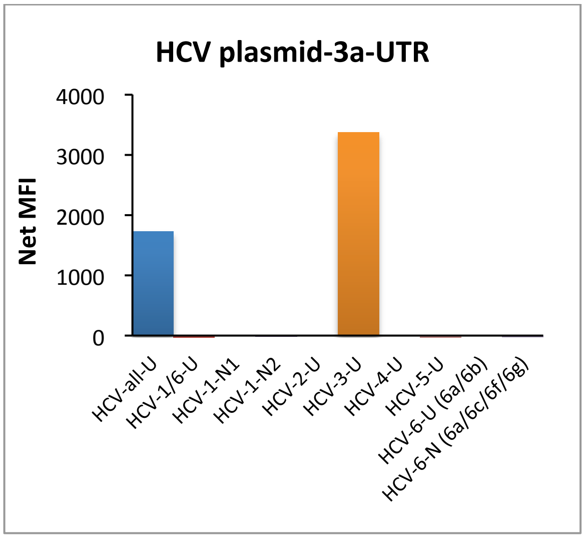
HCV plasmid-3a-UTR
Net MFI
0
1000
2000
3000
4000
HCV-all-U
HCV-1/6-U
HCV-1-N1
HCV-1-N2
HCV-2-U
HCV-3-U
HCV-4-U
HCV-5-U
HCV-6-U (6a/6b)
HCV-6-N (6a/6c/6f/6g)

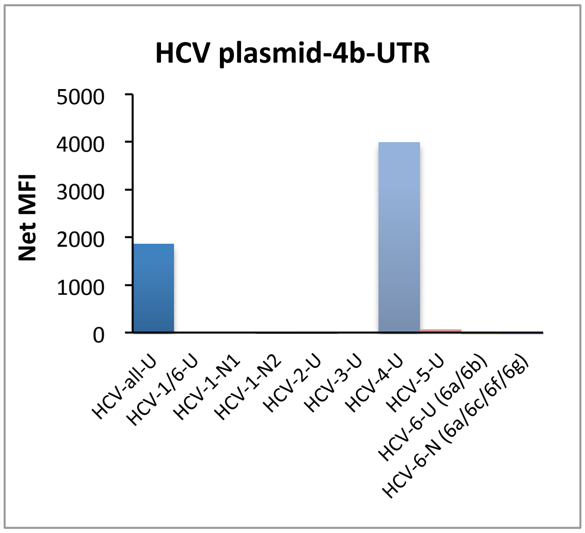
HCV plasmid-4b-UTR
Net MFI
0
1000
2000
3000
4000
5000
HCV-all-U
HCV-1/6-U
HCV-1-N1
HCV-1-N2
HCV-2-U
HCV-3-U
HCV-4-U
HCV-5-U
HCV-6-U (6a/6b)
HCV-6-N (6a/6c/6f/6g)

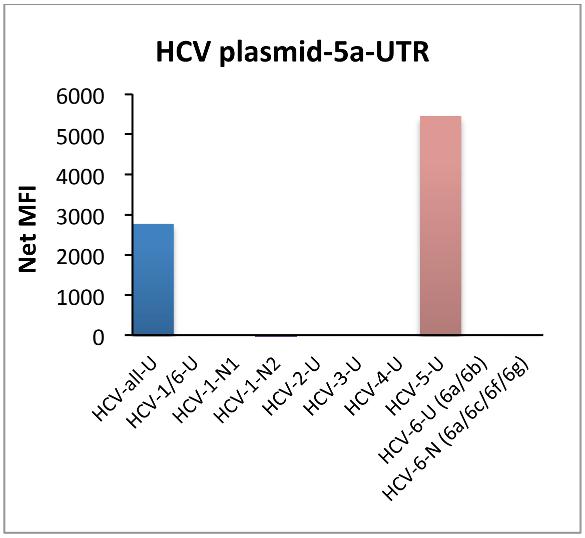
HCV plasmid-5a-UTR
Net MFI
0
1000
2000
3000
4000
5000
6000
HCV-all-U
HCV-1/6-U
HCV-1-N1
HCV-1-N2
HCV-2-U
HCV-3-U
HCV-4-U
HCV-5-U
HCV-6-U (6a/6b)
HCV-6-N (6a/6c/6f/6g)

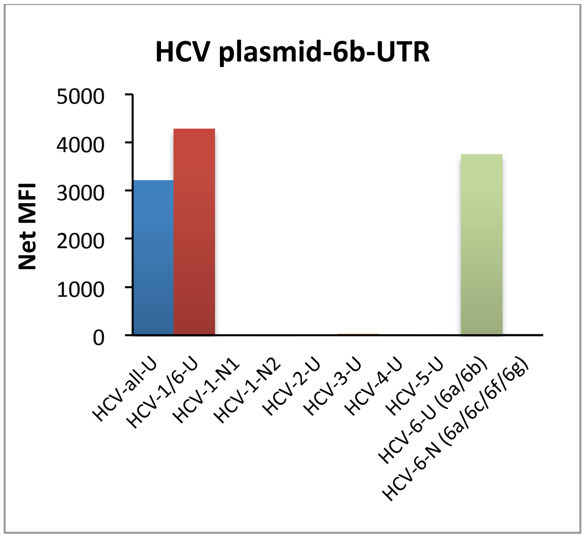
HCV plasmid-6b-UTR
Net MFI
0
1000
2000
3000
4000
5000
HCV-all-U
HCV-1/6-U
HCV-1-N1
HCV-1-N2
HCV-2-U
HCV-3-U
HCV-4-U
HCV-5-U
HCV-6-U (6a/6b)
HCV-6-N (6a/6c/6f/6g)

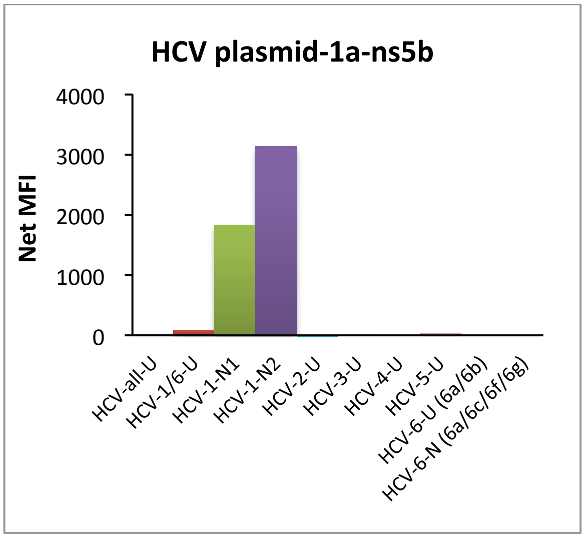
HCV plasmid-1a-ns5b
Net MFI
0
1000
2000
3000
4000
HCV-all-U
HCV-1/6-U
HCV-1-N1
HCV-1-N2
HCV-2-U
HCV-3-U
HCV-4-U
HCV-5-U
HCV-6-U (6a/6b)
HCV-6-N (6a/6c/6f/6g)

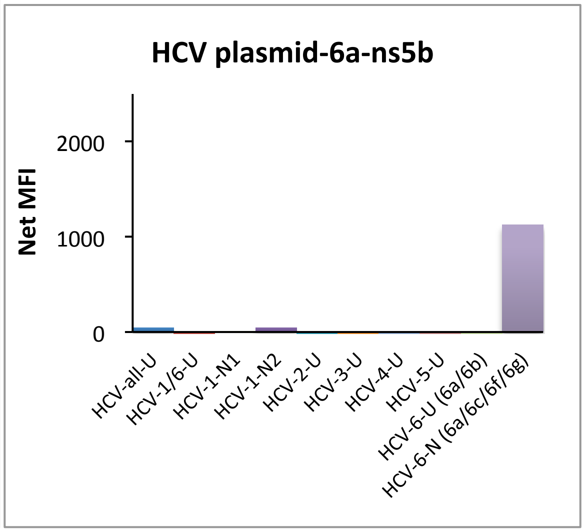
HCV plasmid-6a-ns5b
Net MFI
0
1000
2000
HCV-all-U
HCV-1/6-U
HCV-1-N1
HCV-1-N2
HCV-2-U
HCV-3-U
HCV-4-U
HCV-5-U
HCV-6-U (6a/6b)
HCV-6-N (6a/6c/6f/6g)

(B)

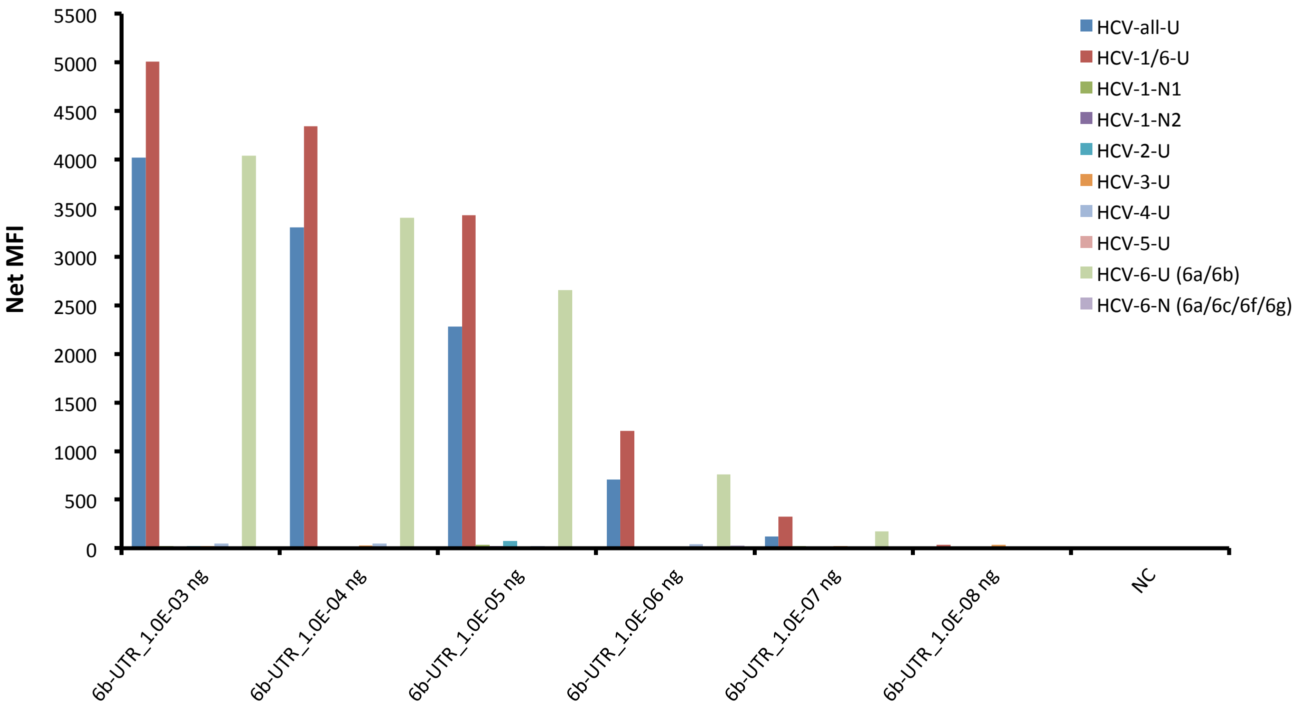

5500
5000
4500
4000
3500
3000
2500
2000
1500
1000
500
0
Net MFI
HCV-all-U
HCV-1/6-U
HCV-1-N1
HCV-1-N2
HCV-2-U
HCV-3-U
HCV-4-U
HCV-5-U
HCV-6-U (6a/6b)
HCV-6-N (6a/6c/6f/6g)
6b-UTR_1.0E-03 ng
6b-UTR_1.0E-04 ng
6b-UTR_1.0E-05 ng
6b-UTR_1.0E-06 ng
6b-UTR_1.0E-07 ng
6b-UTR_1.0E-08 ng
NC

Figure S2.

(A)

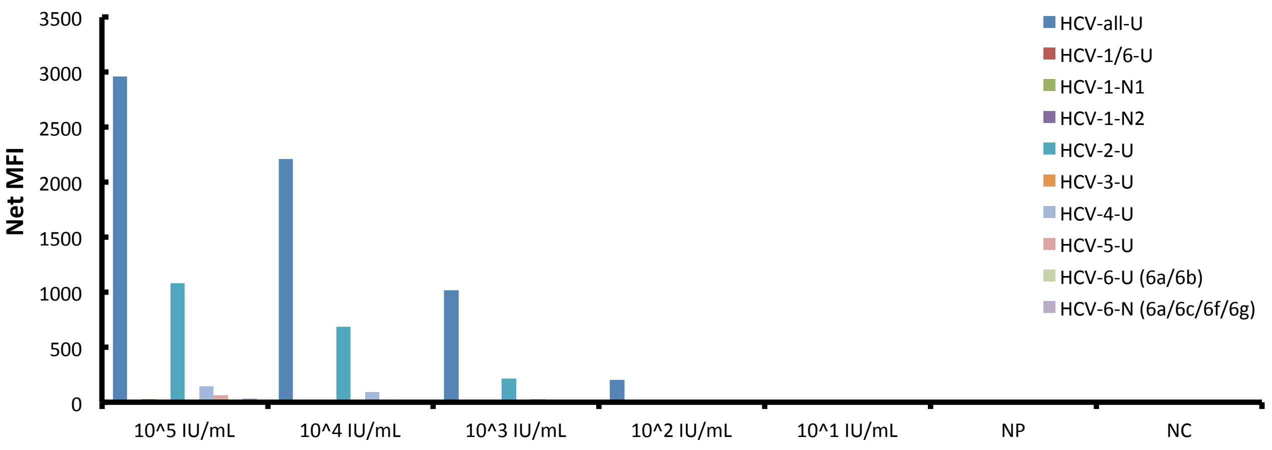

Net MFI
3500
3000
2500
2000
1500
1000
500
0
10^5 IU/mL
10^4 IU/mL
10^3 IU/mL
10^2 IU/mL
10^1 IU/mL
NP
NC
HCV-all-U
HCV-1/6-U
HCV-1-N1
HCV-1-N2
HCV-2-U
HCV-3-U
HCV-4-U
HCV-5-U
HCV-6-U (6a/6b)
HCV-6-N (6a/6c/6f/6g)

(B)

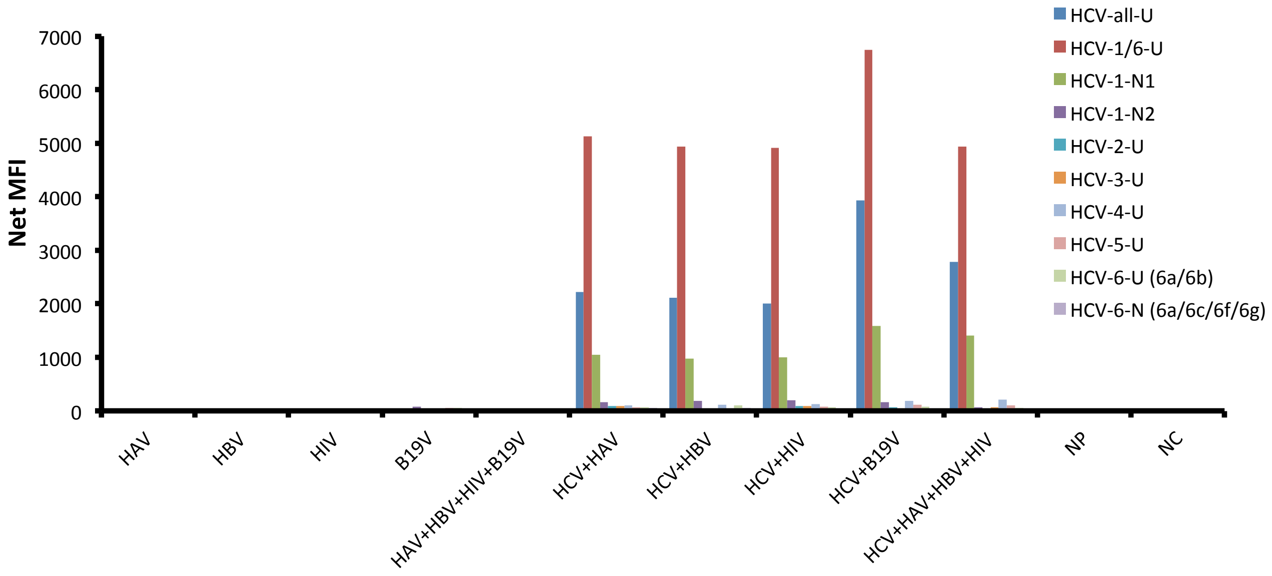

Net MFI
7000
6000
5000
4000
3000
2000
1000
0
HAV
HBV
HIV
B19V
HAV+HBV+HIV+B19V
HCV+HAV
HCV+HBV
HCV+HIV
HCV+B19V
HCV+HAV+HBV+HIV
NP
NC
HCV-all-U
HCV-1/6-U
HCV-1-N1
HCV-1-N2
HCV-2-U
HCV-3-U
HCV-4-U
HCV-5-U
HCV-6-U (6a/6b)
HCV-6-N (6a/6c/6f/6g)
