# Supplementary material for: A reliable multiplex genotyping assay for HCV using a suspension bead array
Source: Microb Biotechnol. 2014 Jul 10;8(1):93–102. doi: 10.1111/1751-7915.12140 (PMC4321376; doi:10.1111/1751-7915.12140)
Supplement: Supplementary file 2 [file mbt20008-0093-sd2.pdf]

Table S1. The primers and target-specific primers used for the HCV genotyping assay.

Primers

Primer name	Sequence (5'-3')	Product size (bp)
HCV-UTR-All-F	CCATGGCGTTAGTATGAGTGT	229 bp
HCV-UTR-All-R	AAGCACCCTATCAGGCAGTA	
HCV-1/6 all-F3	CACACTCCAGTNAAYTC<br>N: A/T/G/C; Y: T/C	277 bp
HCV-1/6 all-R	CCCCAAGTTTCCTGAG	

Target-specific primers

Genotype	Target-specific primer name	Sequence (5'-3')
HCV all	HCV-all-U	Tagged primer: CTTTCTCATACTTTCAACTAATTT-ACCGGTTCCGCAGAC
HCV gt 1 & 6	HCV-1/6-U	Tagged primer: ACATCAAATTCTTTCAATATCTTC-GCACGCCCAAATCTCCA
HCV gt 1	HCV-1-N1	Tagged primer: TTAACAACTTATACAAACACAAAC-TGGAGTGAAAATGCGC
HCV gt 1	HCV-1-N2	Tagged primer: CAAACAAACATTCAAATATCAATC-ATGTTGCCTAGCCAG
HCV gt 2	HCV-2-U	Tagged primer: AATCAACACACAATAACATTCATA-GGGGCACGCCCAAATG
HCV gt 3	HCV-3-U	Tagged primer: TAACTTACACTTAACTATCATCTT-CACTACTCGGCTAGT
HCV gt 4	HCV-4-U	Tagged primer: CATCTTCATATCAATTCTCTTATT-TCCTGGAGGCTGTACA
HCV gt 5	HCV-5-U	Tagged primer: TTAATACAATTCTCTCTTTCTCTA-GGGTCCTGGAGGCTGTT
HCV gt 6	HCV-6-U (6a/6b)	Tagged primer: TCTTACTAATTTCAATACTCTTAC-GCGGGTTTGATCCAAT
HCV gt 6	HCV-6-N (6a/6c/6f/6g)	Tagged primer: CTTAACATTTAACTTCTATAACAC-CGCACCCATAT
